# Supplementary material for: Mesoscale spatiotemporal variability in a complex host-parasite system influenced by intermediate host body size
Source: PeerJ. 2017 Aug 17;5:e3675. doi: 10.7717/peerj.3675 (PMC5563442; doi:10.7717/peerj.3675)
Supplement: Supplemental Information 1 — Preliminary regression between total number of visitors and bird abundance across sites and over time; b parameter and log-log regression between s2 and m and instantaneous correlation between bird abundance/prevalence/aggregation and small and large molecrabs at three month lag across sites. [file peerj-05-3675-s001.pdf]

Supplementary material of the paper entitled

Mesoscale spatiotemporal variability in a complex host-parasite system influenced by intermediate host body size

Sara M. Rodríguez[1, 2] & Nelson Valdivia [2, 3]

[1]Programa de Doctorado en Biología Marina, Facultad de Ciencias, Universidad Austral de Chile, campus Isla Teja s/n, Valdivia, Chile.
[2]Instituto de Ciencias Marinas y Limnológicas, Facultad de Ciencias, Universidad Austral de Chile, canpus Isla Teja s/n, Valdivia, Chile.
[3]Centro FONDAP de Investigación en Dinámica de Ecosistemas Marinos de Altas Latitudes (IDEAL).

Corresponding author:

Sara M. Rodríguez

Email addresses: saramrodriz@gmail.com

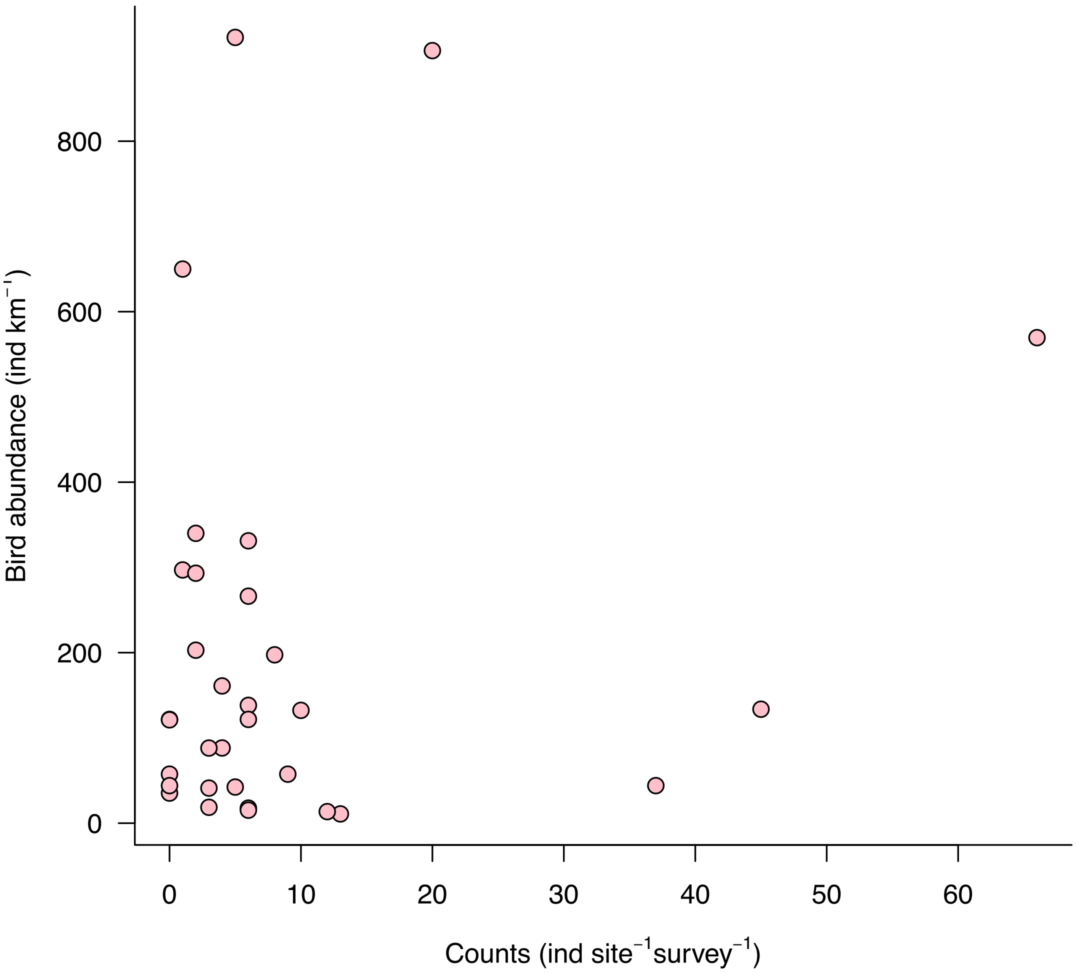

Fig. 1S. Relationships between count of visitors (the sum of number of people, dogs and vehicles; ind site^{-1}survey^{-1}) and bird abundance (ind km^{-1}) across sites and over time. (Generalised mixed-effect model: $R^2m = 0{,}002$, $R^2c = 0{,}673$; $P > 0{,}05$).

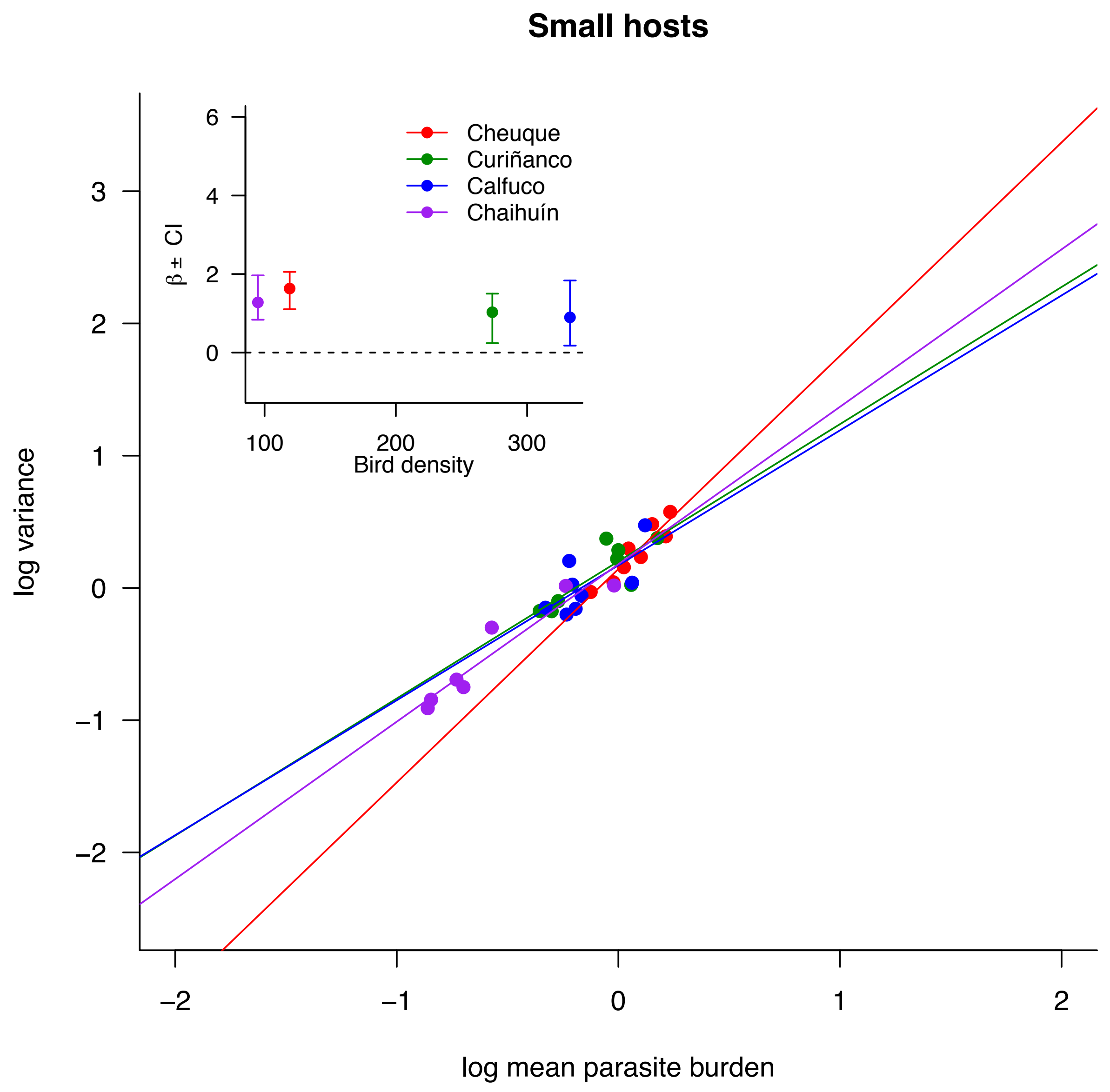

Fig. 2S. Relationships between log variance vs log mean parasite burden in the small molecrabs. Small upper left plot correspond to relationship between *b* (slope) and bird density across sites and over time.

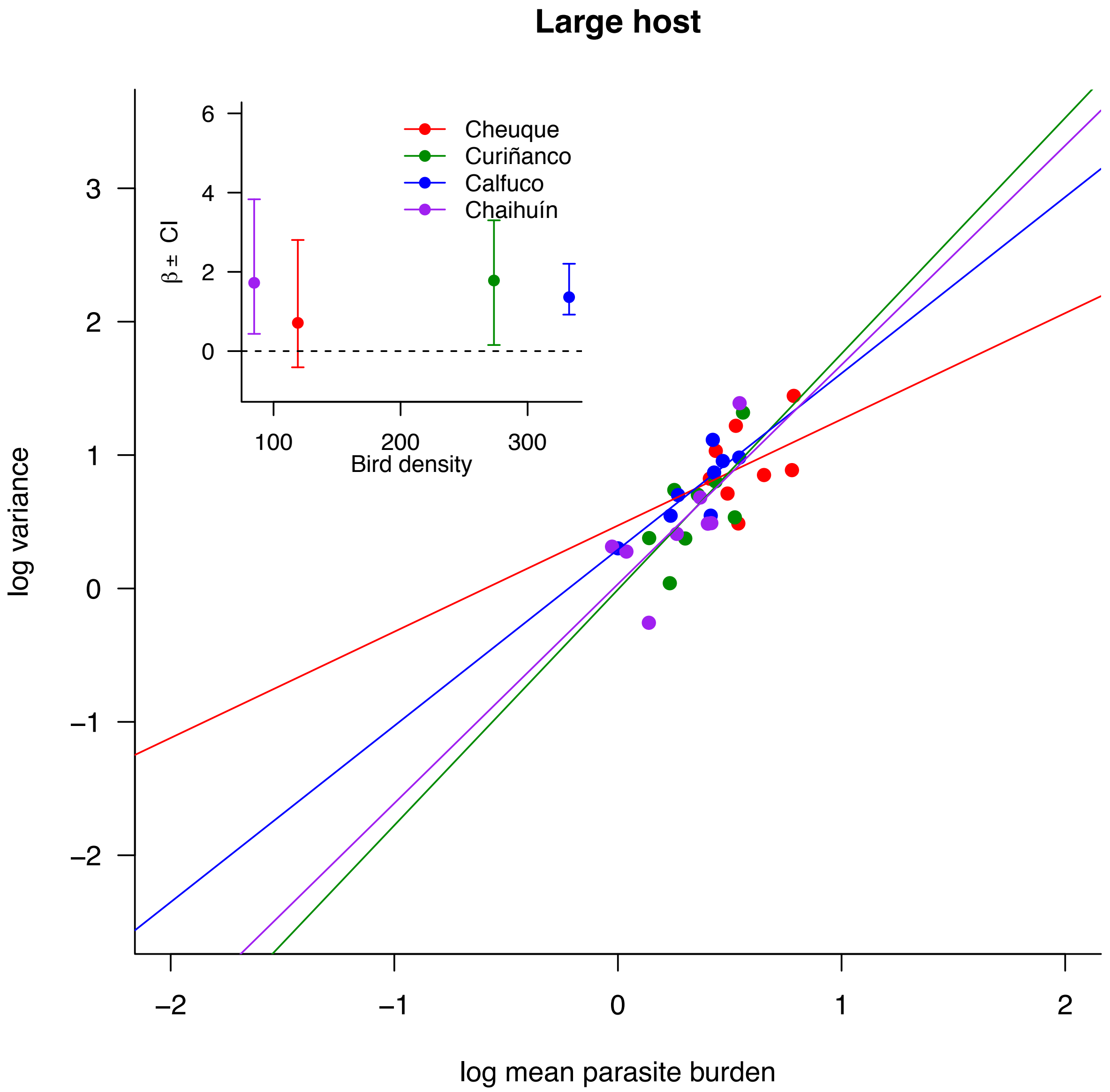

Fig. 3S. Relationships between log variance vs log mean parasite burden in the large molecrabs. Small upper left plot correspond to relationship between *b* (slope) and bird density across sites and over time.

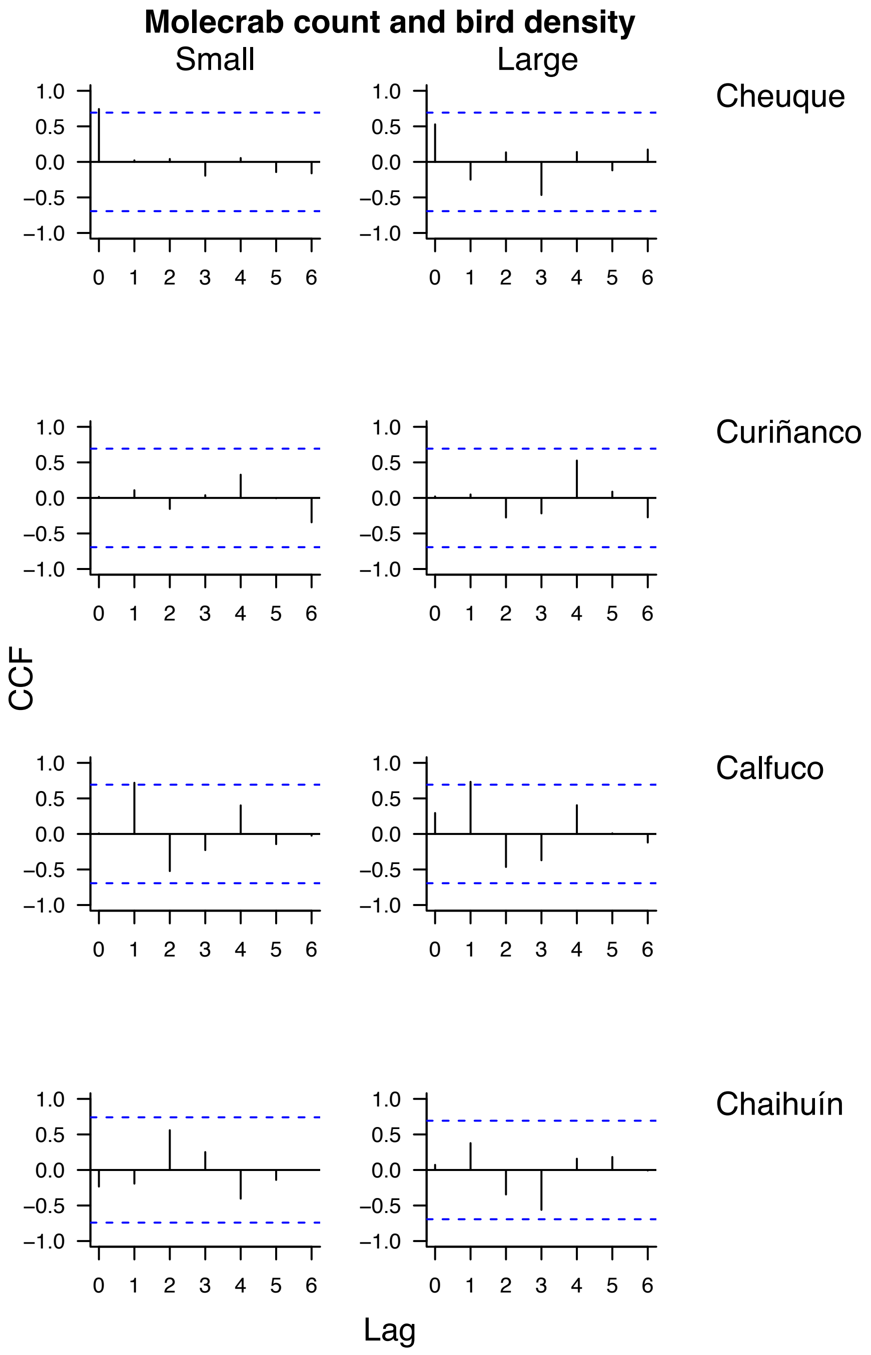

Fig. 4S. Cross-correlation functions (CCF) between sample size (number of hosts) and bird abundance across sites and over time. Each lags number is equivalent to three month.

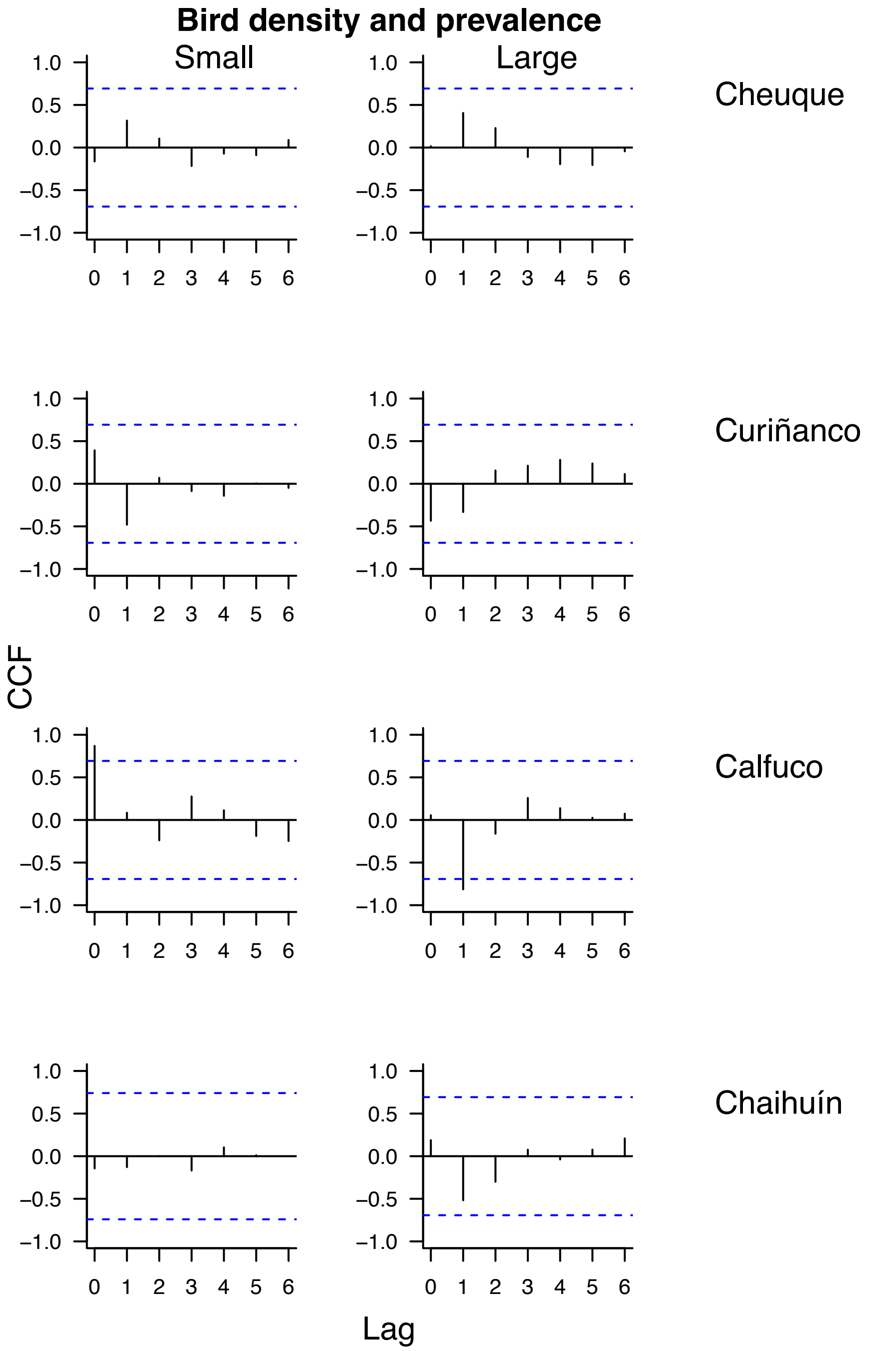

Fig. 5S. Cross-correlation functions (CCF) between bird density and parasite prevalence across intermediate host populations. Each lags number is equivalent to three month.

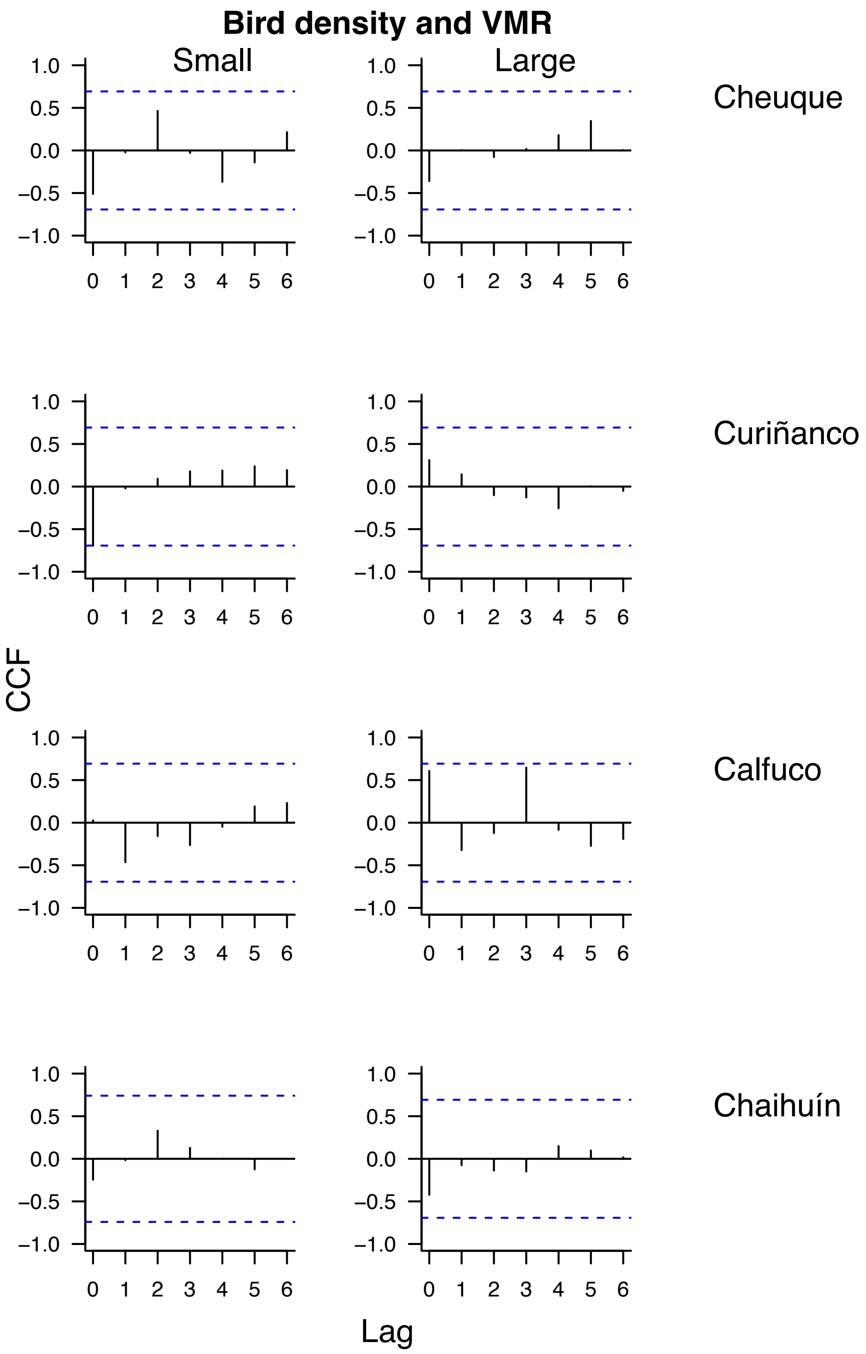

Fig. 6S. Cross-correlation functions (CCF) between bird density and parasite aggregation across intermediate host populations. Each lags number is equivalent to three month.

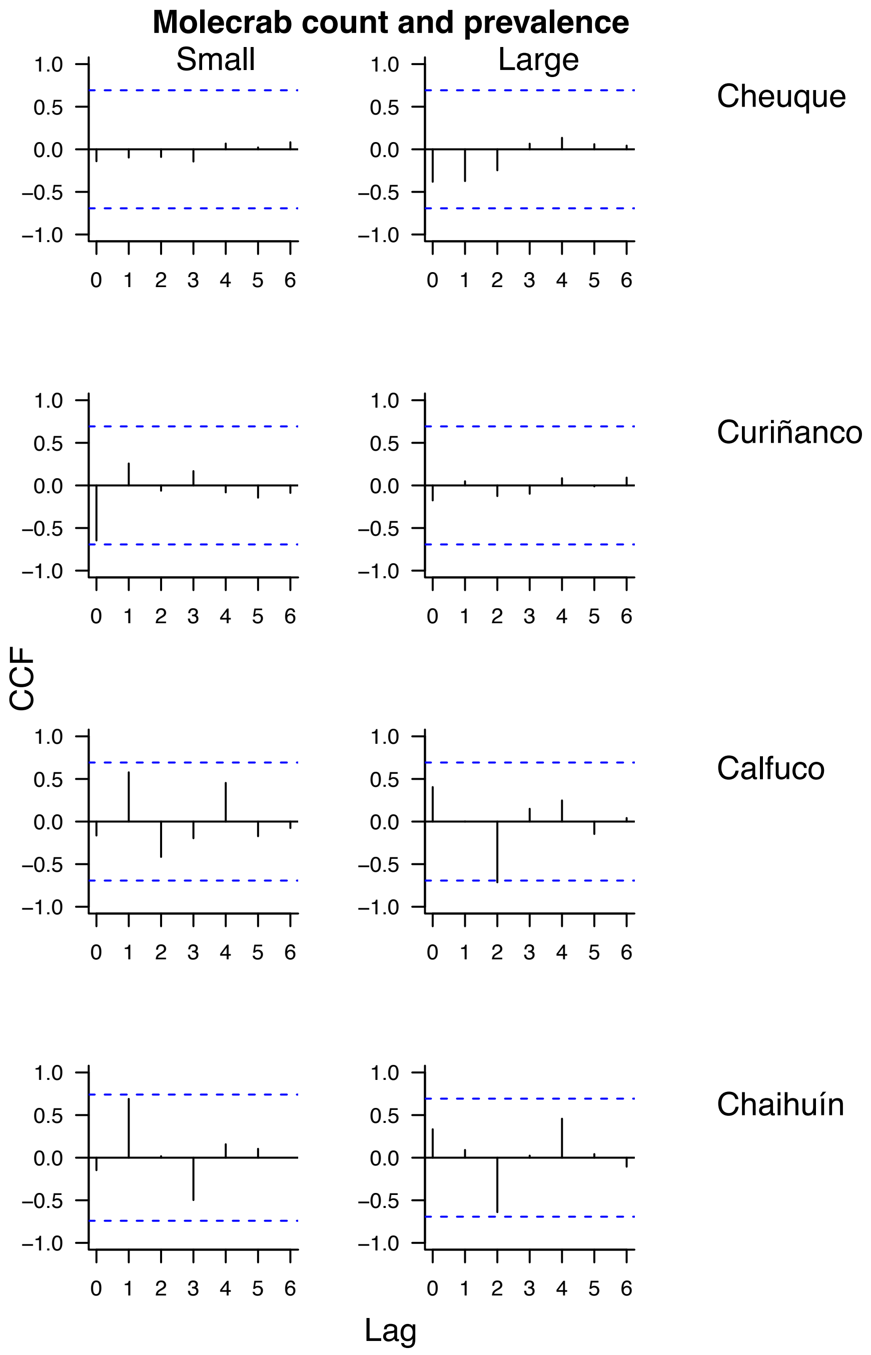

Fig. 7S. Cross-correlation functions (CCF) between sample size (number of hosts) and parasite prevalence across intermediate host populations. Each lags number is equivalent to three month.

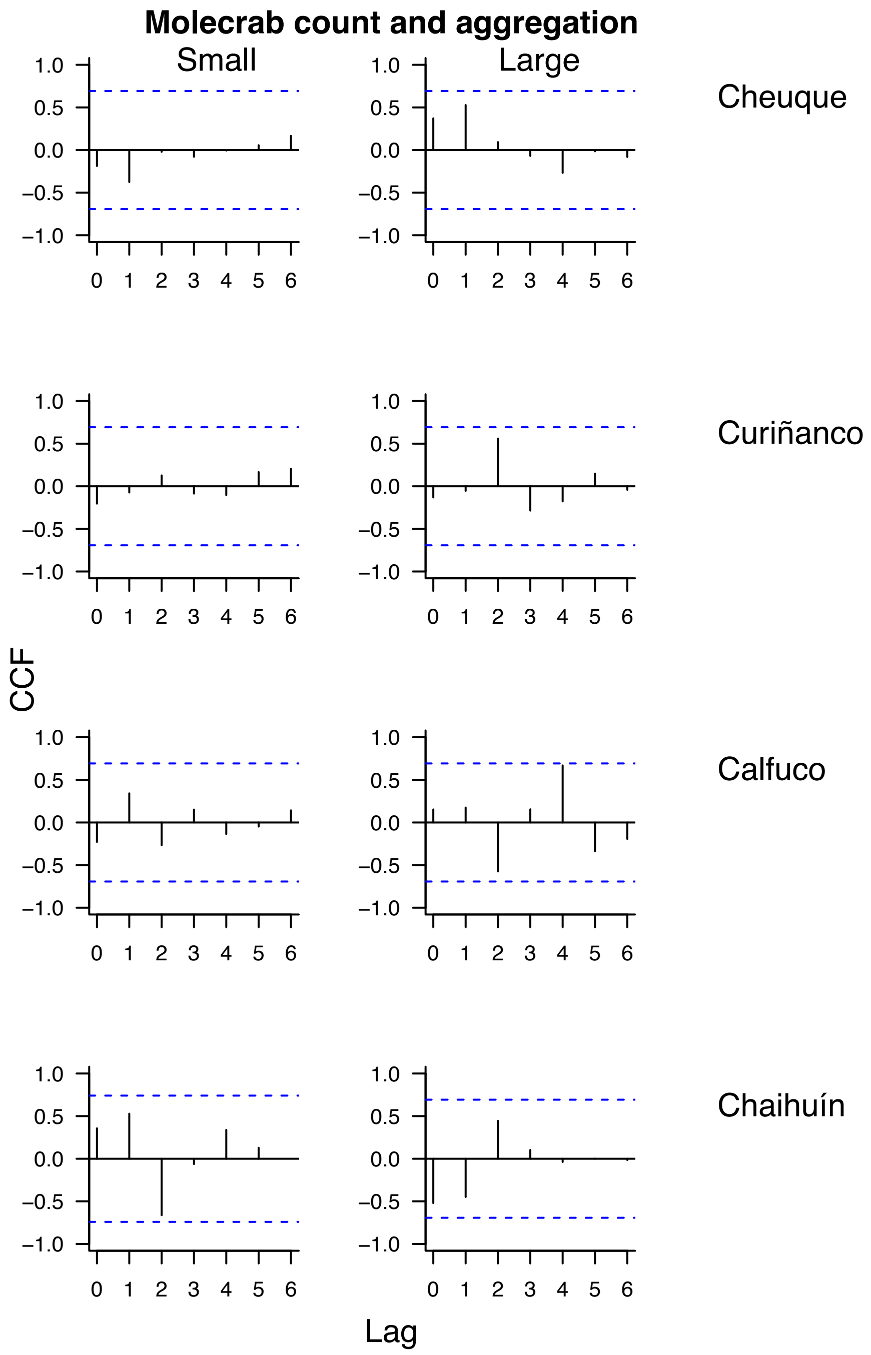

Fig. 8S. Cross-correlation functions (CCF) between sample size (number of hosts) and parasite aggregation across intermediate host populations. Each lags number is equivalent to three month.
