# Supplementary material for: Mesoscale spatiotemporal variability in a complex host-parasite system influenced by intermediate host body size
Source: PeerJ. 2017 Aug 17;5:e3675. doi: 10.7717/peerj.3675 (PMC5563442; doi:10.7717/peerj.3675)
Supplement: Supplemental Information 3 — Preliminary analyses between standardized residuals and fitted values of prevalence vs. bird density and host size and auto-correlation functions between prevalence/aggregation (variance-to-mean ratio) vs. bird density and host size across sites and over time. [file peerj-05-3675-s003.pdf]

Supplementary material of the paper entitled. Only for reviewers.

Mesoscale spatiotemporal variability in a complex host-parasite system influenced by intermediate host body size

Sara M. Rodríguez[1, 2] & Nelson Valdivia [2, 3]

[1]Programa de Doctorado en Biología Marina, Facultad de Ciencias, Universidad Austral de Chile, campus Isla Teja s/n, Valdivia, Chile.

[2]Instituto de Ciencias Marinas y Limnológicas, Facultad de Ciencias, Universidad Austral de Chile, canpus Isla Teja s/n, Valdivia, Chile.

[3]Centro FONDAP de Investigación en Dinámica de Ecosistemas Marinos de Altas Latitudes (IDEAL).

Corresponding author:

Sara M. Rodríguez

Email addresses: saramrodriz@gmail.com

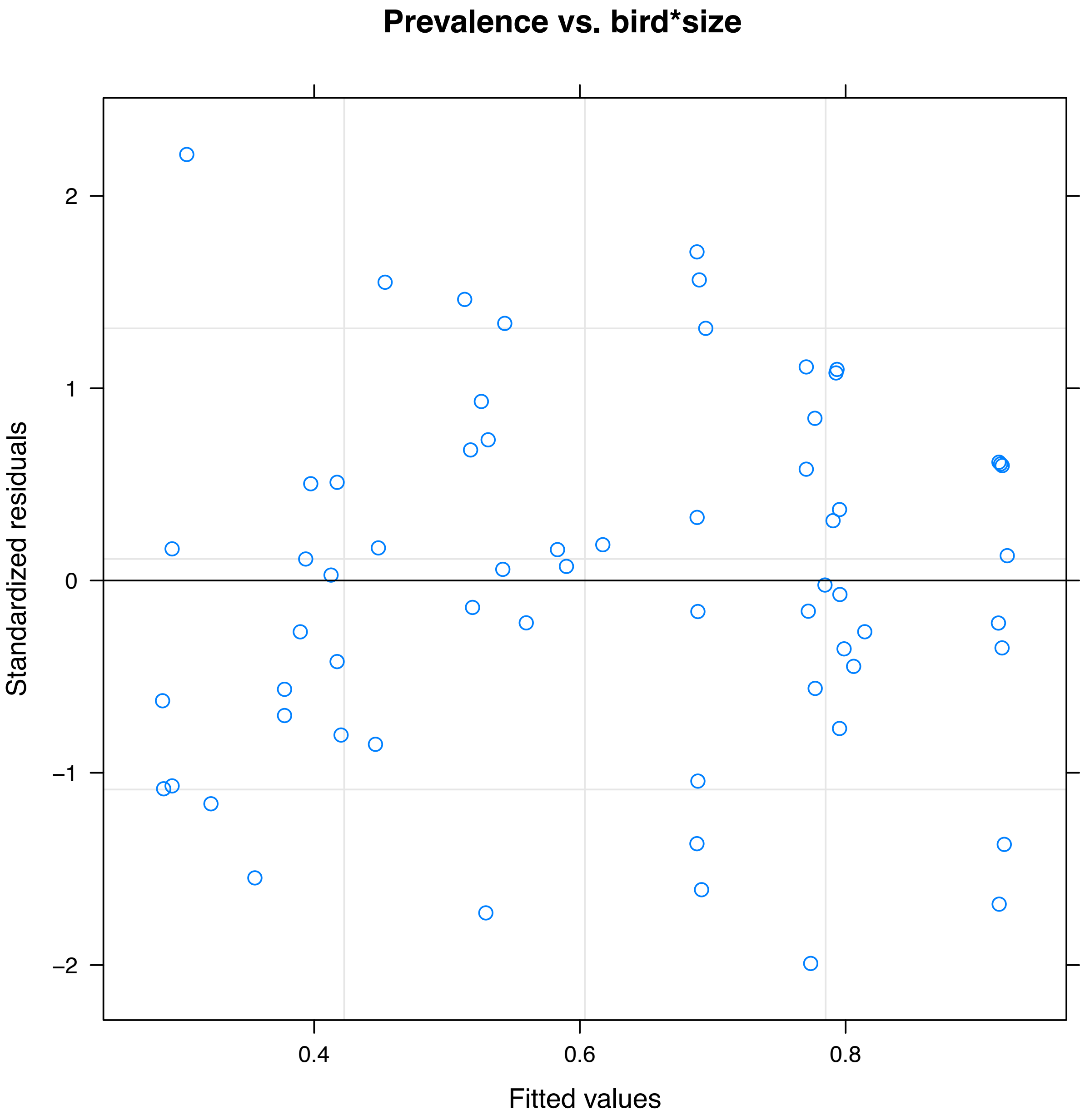

Fig. 1. Relationships between bird density and host size effects on prevalence of parasites.

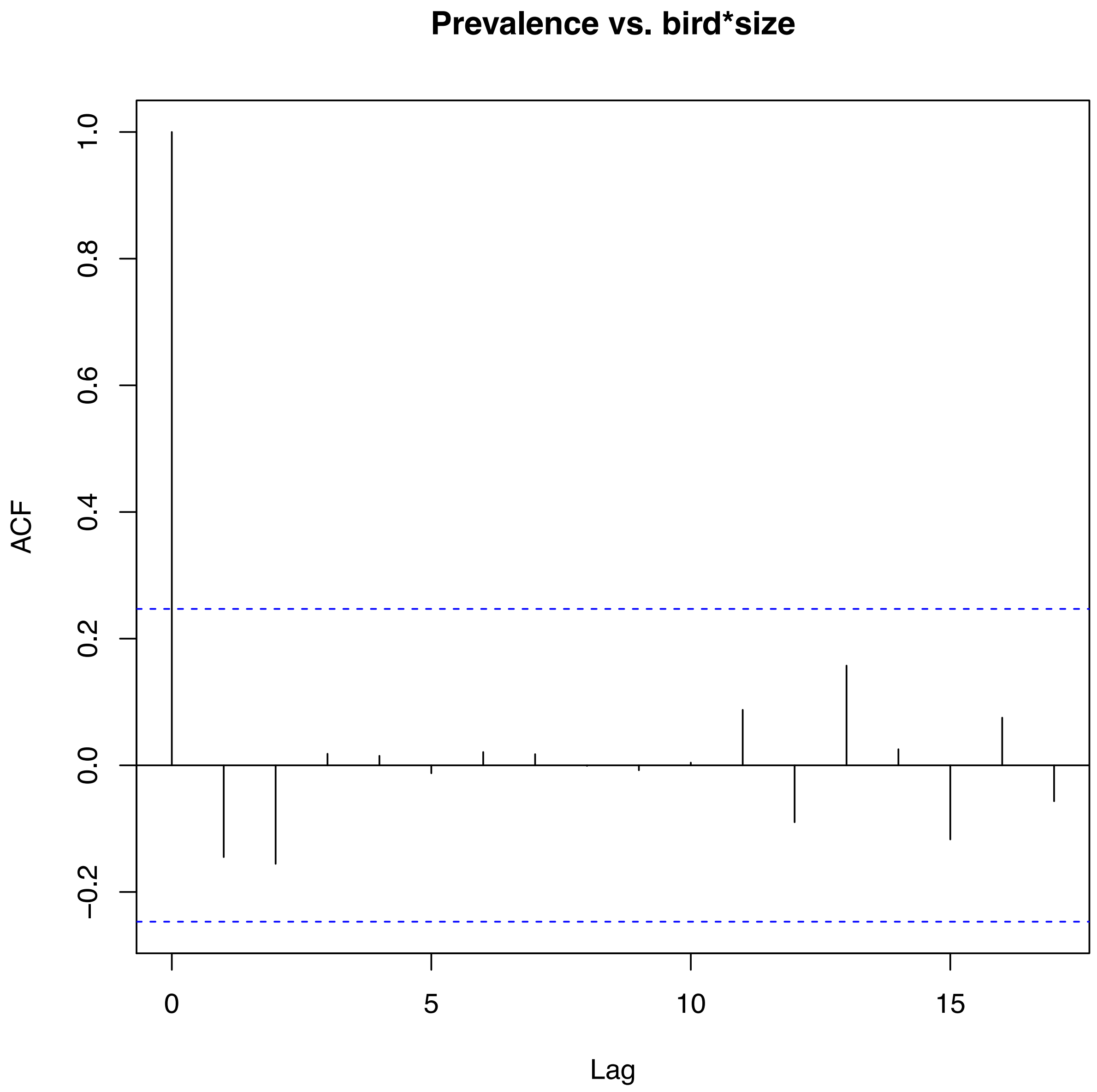

Fig. 2. Auto-correlation functions (ACF) between prevalence vs. bird density and host size across sites and over time.

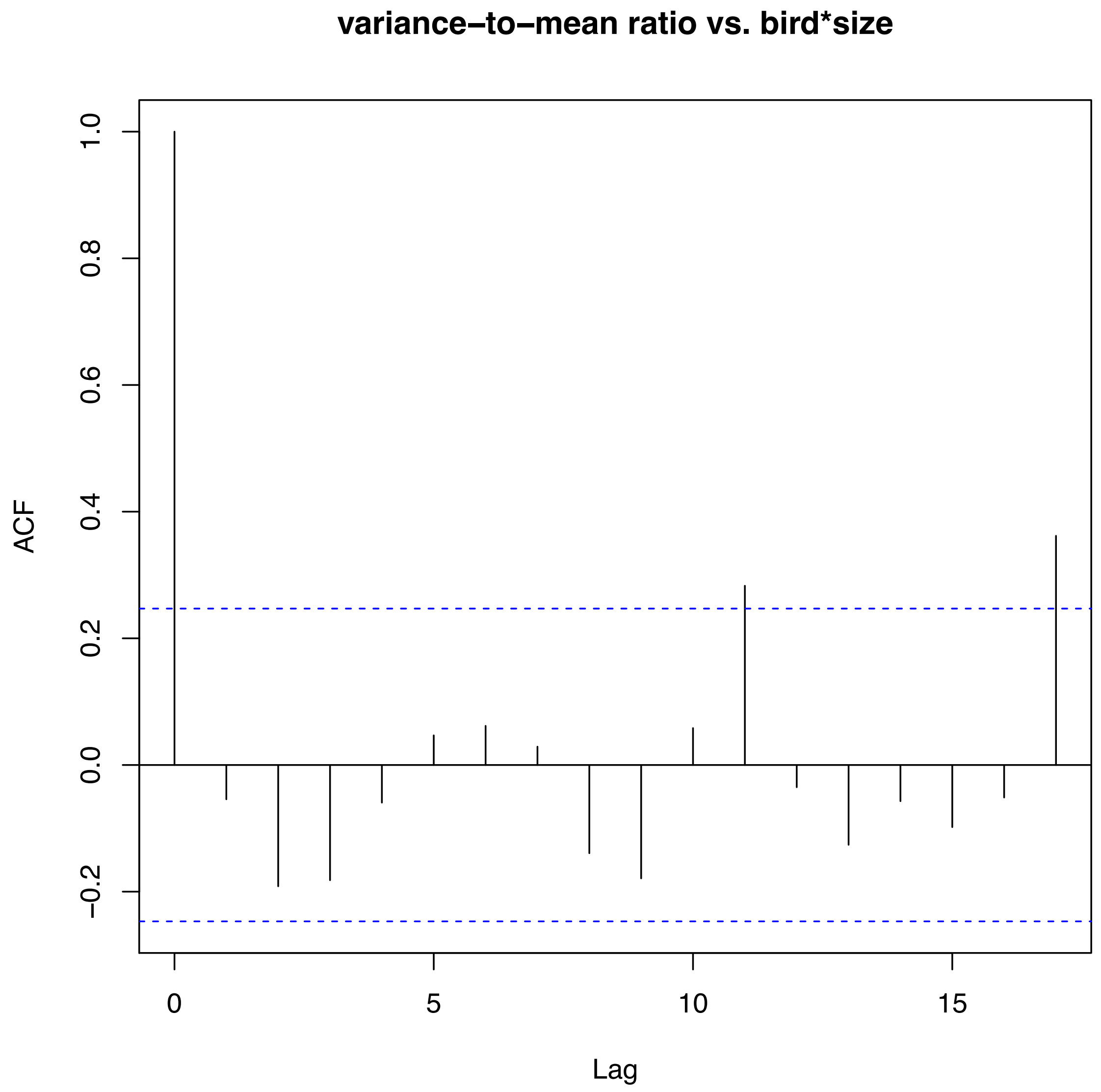

Fig. 3. Auto-correlation functions (ACF) between variance-to-mean-ratio vs. bird density and host size across sites and over time.
